# Ten simple rules to initiate and run a postdoctoral association

**DOI:** 10.1371/journal.pcbi.1005664

**Published:** 2017-08-17

**Authors:** Chiara Bruckmann, Endre Sebestyén

**Affiliations:** 1 IFOM, the FIRC Institute of Molecular Oncology, Milan, Italy; 2 IPA (IFOM Postdoc Association), IFOM, the FIRC Institute of Molecular Oncology, Milan, Italy; Whitehead Institute, UNITED STATES

## Introduction

Postdoctoral researchers (postdocs) are individuals who have obtained a PhD degree and are working in a temporary, mentored research position [1,2]. A proper postdoctoral experience should provide the necessary training for these individuals to achieve scientific and professional independence. During this phase of researchers’ careers, they should develop new skillsets by working in research groups. At the same time, they are usually involved in multiple tasks, such as mentoring trainees, applying for grants, writing publications, and keeping up with the latest advances in their field [3,4]. As young researchers enter the late-PhD and post-PhD period, they apply for positions with mentors whose work interests them. Mentors help postdocs prepare to lead research groups and improve existing skills or develop new ones [5].

In science, the number of researchers holding a doctoral degree and looking for postdoc positions has doubled in less than 2 decades [4]. However, the number of new academic jobs has not kept pace with this increase. Despite their restricted career prospects, stemming from the highly competitive environment they face [4,6–9], most postdocs wish to remain in research, and nonacademic career paths are often seen as failure [8,10–14]. At the same time, specific training that emphasizes lab management or transferable skills, opportunities to transition into alternative career paths, and coaching on complementary skills are scarce [15,16]. As early-stage researchers are often encouraged to broaden their experience through a stay abroad [2], a postdoctoral position is frequently accompanied by a geographical transition, along with all the challenges of living in a different country and leaving circles of friends and networks of colleagues [17,18].

Postdoctoral associations can improve the quality of the postdoctoral experience and provide support for professional development and everyday working life. Unlike graduate students, postdocs do not have classmates to whom they can turn for support and networking; therefore, a structure that facilitates their integration into their institution can instantly improve their quality of life. As postdocs pay neither tuition nor student fees, institutions dedicate substantially fewer resources to them, even though they constitute a large fraction of the labor force and, together with PhD students, are the major engine driving research. Therefore, a postdoc association can contribute significantly to the professional development of postdoctoral researchers by organizing networking and career events, thus widening their prospects [19]. An established postdoctoral association can facilitate open communication with the host institution and administrative bodies while also helping to develop a nurturing research environment. Additionally, active participation in an association will help postdocs prepare for leadership roles outside the lab and strengthen their curricula vitae (CVs) [20]. At present, only a small number of postdocs spend time developing additional skills beyond research skills during their postdoctoral period.

A postdoctoral association is beneficial not only for the postdocs but also for the hosting institution, as clear communication and representation of postdoc problems or needs leads to a better work environment. Top-level research is just a part of the puzzle that attracts talent to an institution. A positive work environment is self-reinforcing: open communication and internal networking support one another, and together, they create an environment that is creative, fun, hardworking, and highly productive [21].

In the United States, postdoctoral associations are present in many research institutes and universities, and the National Postdoctoral Association (NPA) organizes an annual conference, besides providing resources for postdocs and (where present) for postdoctoral offices [1]. However, there is little central coordination of different local associations. In the rest of the world, postdoc associations are sporadic and even more isolated than in the US. By sharing our experience in starting and running a postdoc association, and the challenges and joys we encountered, we wish to encourage postdocs to start other postdoc associations.

To start and run a postdoctoral association, we suggest the following 10 basic steps.

## Rule 1: Recruit a handful of motivated postdocs interested in starting a postdoc association

Call a meeting and advertise it by putting up flyers throughout research buildings, seminar rooms, libraries, and cafeterias and by talking directly to your own colleagues. Expect only a handful of attendees—but those who will show up will most likely express interest in setting up a postdoctoral association. Define the general aims of the association. The initial mission of a postdoc association can be very generic at first—for example, to enhance scientific interactions among postdocs and improve the quality of their personal life. Later, it can be tailored for the specific needs of your institution.

## Rule 2: Work together with other entities within the institution, such as human resources and senior administration

Links with administrators in graduate and postdoctoral offices, human resources, communications, grant management, and international offices are fundamental. It is important to facilitate discussions between postdocs and other entities within the institution in order to make the community more inclusive and integrated and to encourage collaboration and sharing of ideas. The postdoc association can work together with administrators, for example, to gather data on postdoc needs, keep an up-to-date email list of all postdocs, establish an alumni database of graduates who are working scientists, and set up a common calendar of events. This facilitates internal institutional networking and promotes personal and scientific relationships between present and past researchers of the institution [22]. Ideally, every institution should create a postdoctoral service office or similar unit (run by permanent employees) to keep postdoctoral researchers engaged in planning their careers and foster programs for their benefit [23]. The dearth of such postdoctoral offices and representatives is a significant reason why the challenges and problems of postdoc life remain practically invisible. If creating a postdoctoral office is not feasible (for example, if an institution does not have enough postdocs), the institution could consider giving the responsibility of supervising postdoctoral researchers to an existing administrative office—for example, to the graduate office. Participation in university committees and regular meetings with the relevant dean or the institution’s chancellor or director of the institution are important opportunities for discussing postdoc issues and representing postdoc interests.

## Rule 3: Call for official elections of the association board and write statutes

Approximately 5–10 representatives, meeting monthly, should constitute the board of the association. The representatives should be elected by postdoctoral researchers themselves, and their mandate should be annual, as postdocs are often hired for 1 or 2 years. Nominees should include postdocs from multiple departments in order to guarantee diversity and the expression of various interests. After its election, the board should elect a president and a vice-president. Besides these 2 roles, in our experience, it might be difficult to define a specific structure for young associations. In the beginning, roles and responsibilities should be flexible, as postdocs come and go and also need to deal with their own deadlines and core responsibilities as researchers. When the association is more mature and well established within the institution, and when successful generational turnover of the postdoctoral representatives is therefore supported, you can define clearer structures, procedures, and roles for officers or board members [24]. As the workload for active postdoctoral representatives can be heavy, even when convening to address administrative matters, it is advisable to try avoiding meetings that last for more than 1 hour so that people are able to return to their other pressing duties. If the institution has a high percentage of international postdoctoral fellows, the board should be required to encompass representatives from different nationalities, thus gathering diverse views and opinions. Bear in mind that even an experienced postdoctoral association will face problems with issues such as the recruitment of new representatives and overcoming generational change, and this is particularly true for a newborn association. A successful strategy for volunteer recruitment should include extensive advertisement (both physical and electronic, for example, via a postdoc email list), active networking at social events and initiatives, and practical support on advertisement from administrative and human resource offices. The statutes of the postdoctoral association should be clear, concise, and distributed together with the aims of the association, by the human resource office to every new postdoc joining the institution, as part of a welcome package.

## Rule 4: Listen to the needs of your colleagues by running regular surveys and organize events to respond to different needs

As a first initiative of the newly founded postdoctoral association, organize a short meeting, inviting all postdocs, and distribute an anonymous questionnaire to help you understand their needs in your institution. The survey could include detailed questions on gender, dependents living with postdocs, satisfaction with salaries or working conditions, and long-term career goals (inter alia). All of this information will help in planning more useful events and understanding postdoc life generally. One’s colleagues may not just want to get classic scientific training but may be eager to learn complementary skills, create connections to local companies, or just put together sport tournaments among colleagues. As a general rule, tailor the location, timing, and content of events to postdoc needs so that they are motivated to attend. For example, these events should be organized on or near campus, around lunchtime, at the end of working hours, or at another convenient time; this way, people can show up for an hour and then return to work or go home.

## Rule 5: Provide support for international postdocs

Many researchers move abroad for their postdoctoral research; therefore, they can feel out of place and isolated in an unfamiliar culture and can find it difficult to integrate socially as they spend most of their time working (a vicious cycle). If your institution lacks an international or diversity office, provide support for international postdocs yourselves and ensure that their particular needs are addressed. This means connecting with administrative and human resource personnel who can help in integrating newcomers. A postdoctoral association can support international postdocs by offering international lunches or coffee hours or by promoting the culture of the hosting country, for example, by organizing visits to exhibitions or restaurants. Together with the administration, the association should also provide language courses. Institutions that ensure social integration of postdoctoral fellows are more easily able to attract talent from abroad, which means this should also be a priority from an institutional perspective. A description of the association should be present on the institution’s website, together with information on past events.

## Rule 6: Organize and promote career development events

Organize career events and professional development courses for postdocs and help to educate postdocs on the transferable skills they have that will be useful for promoting their careers both inside and outside academia. Various companies run tailored workshops for transferable skill development or project management. Unfortunately, not all institutions can afford to outsource this kind of training; therefore, while looking for an external sponsor (see Rule 10), the postdoc association, together with the postdoctoral office, can take advantage of internal know-how. For example, you could recruit principal investigators for mock interview workshops in which they explain the application path for academic positions and give tips and suggestions for successful proposals, CVs, and interviews [25]. In addition, you could invite successful alumni pursuing careers outside of classic academic research to share their personal career paths and lessons. Many postdocs do not know where to find nonacademic career options or how to prepare for them. People who have experienced such situations can advise on choosing next career steps, considering not only the responsibilities of positions and postdocs’ desired salary ranges but also their desired lifestyles. These meetings enable postdocs to talk about their career plans, do professional development activities, and ideally, network with scientists who have completed a PhD but moved outside of academia into the domains of industry, scientific publishing, technical sales, start-ups, teaching and academic support, and so on.

## Rule 7: Get visibility in your institution, using social networks, newsletters, and flyers

Every postdoctoral researcher joining the institution should automatically become part of the association and the postdoc mailing list and thus should be regularly informed about ongoing events and programs. In addition to the mailing list, the association should promote events using a periodic newsletter and flyers. If possible, there should be dedicated spaces in high-traffic areas for posters, flyers, and information on the association. A dedicated website, linked to social media such as LinkedIn, Facebook, and Twitter, should include a short description of the aims of the association, the profiles of the elected representatives, and past and upcoming events. This can be used by the association to disseminate information to current and past postdocs and additionally gives geographically unrestricted visibility, facilitating networking with other associations. Furthermore, postdocs considering moving to the host institution can get in touch through social media or the website and get a sense of the local community.

## Rule 8: Build a community by organizing social events

Balance scientific events with social events. Encourage networking and exchange of ideas by organizing monthly meet-up events for postdocs in an informal setting, such as dinners out, visits to exhibitions/museums, seasonal barbecues, or sport groups (see also Rule 5). These activities can create a supportive peer group and integrate postdocs, helping members socialize, make friends, and discuss work or career steps.

## Rule 9: Network with other postdoctoral associations at the national and international levels

In the US, postdoctoral associations are well established in many institutions and give a voice to the postdoctoral community. These associations are supported by the NPA if they apply for membership. In the rest of the world, postdoctoral associations are not coordinated at any level and usually work in isolation; however, even regional coordination between associations can increase the visibility of the postdoctoral community and facilitate access to training, networks, and events. Some examples include the Postdoctoral Association of the European Molecular Biology Laboratory (EMBL), which is active in 5 cities and 4 countries [26], or the Marie Curie Fellowship Association [27]. However, the former is still limited to 1 institute, while the latter is restricted to Marie Curie Fellowship winners. Connections with other associations may serve as the basis for shared projects such as joint career events, international symposia, and joint funding applications.

## Rule 10: Get sponsored by companies

In order to support large events, you could try to attract a broad range of sponsors and donors such as vendors or industry professionals in different fields of science. For example, you can offer different sponsorship packages, agree to distribute products or company-specific information to event participants, place company logos and links on event websites, or set up promotional booths in event exhibition areas (although you should first check institutional policies, which might limit promotional opportunities or the types of events that can be funded). Vendor fairs and scientific talks given by company representatives are some examples of sponsored events that postdoc associations can use for fundraising. By reaching out directly to participants, sponsoring companies will benefit through advertisement and increased visibility, while postdocs will benefit from the opportunity to hear highly qualified speakers. Group leaders or alumni members who are moving or have moved to industry can provide an extended network of contacts that the association can approach. Companies might also be willing to provide courses by involving their internal human resources and training teams in exchange for the visibility and networking they will garner.

## Conclusions

Postdoctoral researchers are often isolated, their careers at the mercy of their principal investigators or advisors. Unlike students, their affiliation is often not to a department but rather to a specific lab. Establishing a postdoctoral association can be a way to foster networks needed for effective support; however, setting up and running a postdoctoral association requires time commitment from active members and so should ideally be promoted by the institution as part of the postdoc experience.

The need for networking and support among postdocs is strong, but getting people involved in initiating a postdoctoral association might nevertheless be seen as a major obstacle. However, based on our experience, we can say that it is feasible and rewarding. Our hope is that after reading this article, many postdoctoral researchers will feel empowered to start a postdoctoral association at their home institutions. Given the temporary nature of the postdoc, it might take multiple generations to get an association running at full speed, but this should not discourage its founders from taking the initiative and making this important contribution. A positive postdoctoral experience should include establishment of a solid network of colleagues. Accordingly, institutions should motivate postdoctoral fellows to stay in touch with one another and to continuously expand their networks in the present and future.

Finally, we encourage existing postdoctoral associations to network at the national and international levels, taking as an example the NPA in the US. There are a few independent postdoc associations in the European Union that would greatly benefit from coordination with the aim of setting up scientific meetings, networking, applying for common funding for advanced training, and gathering information on postdoctoral lives, career prospects, and needs. As there is no census of existing postdoctoral associations in Europe, and only a small number of reports discuss working conditions, salaries, gender inequality, training, or institutional support, networking would yield excellent opportunities to start collecting this information.

The IFOM (Fondazione Italiana per la Ricerca sul Cancro [FIRC] Institute of Molecular Oncology) Postdoc Association is a newborn association based at IFOM, Milan (Italy), and it would be exciting to see it grow through networking with other Italian and European postdoctoral associations.
